# Induction or aggravation of other immune-mediated disorders by disease-modifying therapy in treatment of multiple sclerosis

**Published:** 2018-07-06

**Authors:** Seyed Mohammad Baghbanian, Mohammad Ali Sahraian

**Affiliations:** 1 Bualicina Hospital, Mazandaran University of Medical sciences, Sari, Iran; 2 Multiple Sclerosis Research Centre, Sina Hospital, Tehran University of Medical Sciences, Tehran, Iran

**Keywords:** Interferon-Beta, Glatiramer Acetate, Autoimmune Disease, Multiple Sclerosis

## Abstract

Interferon beta (IFN-β) and glatiramer acetate (GA) are the primary therapeutic immunomodulatory agents that interfere with relapsing-remitting multiple sclerosis (RRMS), and the most commonly-used drugs as well. Induction or aggravation of other immune-mediated diseases has been reported following INF-β administration. We have reviewed the reported cases to notify the treating physicians about these rare adverse events. Although co-morbid autoimmune disorders have been reported in patients with MS, the pro-inflammatory role of disease-modifying drugs, especially INF-β, could affect and enhance this co-occurrence. Clinical or laboratory autoimmunity histories suggest the use of GA over INF-β as the treatment of choice.

## Introduction

Multiple sclerosis is an inflammatory demyelinating disease of the central nervous system which, in over 85% of circumstances, initially presents as relapsing neurological disability followed by remission phases.^1^^-^^3^ The disease affects young adults and is more common among women.^4^ There is no cure for MS, and treatment concentrates on treating relapses, diminishing the progression of disability and secondary symptom management. Interferon beta (IFN-β) and glatiramer acetate (GA) are the primary therapeutic immunomodulatory agents that interfere with relapsing-remitting multiple sclerosis (RRMS), and the most commonly-used drugs as well.^5^

Isaacs and Lindenmann,^6^ the discovers of INF, use this term to explain the antiviral replication properties of these biologically-active substances. It features an acceptable risk profile, is well tolerated, and is effective in decreasing the activity of RRMS. This makes INF the first choice for disease-modifying therapy in MS.

GA is a synthetic polypeptide composed of four amino acids in a mixture resembling myelin basic protein.^7^ Many trials have shown that GA can reduce the relapse rate, disability progression and ameliorate the course of the disease as indicated by MRI findings.^8^ GA is one of the most prescribed disease-modifying-therapies for multiple sclerosis.^9^

Injection site reaction is the most frequent side effect of IFN and GA. Other side effects include a flu-like syndrome, lymphopenia caused by IFN as well as hepatic failure, hepatitis and elevated liver enzymes. These have been reported for both treatments, although they are more common for IFN.^10^ Induction or aggravation of other immune mediated diseases have been reported following INF beta administration. Here we have reviewed the reported cases to notify the treating physicians about these rare adverse events. In many instances continuation of INF following appearance of a new autoimmune disease may aggravate the condition and INF therapy should be promptly stopped to prevent further damage.

## Review of case reports

***Arthritis:*** A 37-year-old woman with MS presented with isolated *t*emporomandibular joint arthritis two weeks after starting IFN-β1a therapy. It resolved completely shortly after IFN discontinuation, and did not recur after switching treatment to GA.^11^ A 38-year-old woman with RRMS presented with left pre-patellar bursitis with no particular involvement three months after initiation of IFN-β1a.^11^ A 29-year-old woman with RRMS presented with severe acute-onset arthralgia, swelling of both knees, and inflammatory synovial fluid reaction three weeks after beginning treatment with IFN-β1b. The condition disappeared a few days after discontinuation of IFN.^12^ Seropositive polyarthritis with high erythrocyte sedimentation rate (ESR), elevated C-reactive protein (CRP), and high rheumatic factor (RF) titer was reported in a 42-year-old woman with RRMS after 30 months of treatment with INF-β1b.^12^ Animal experiments have shown that GA could exacerbate autoimmune arthritis. These findings suggest that GA should be used with great caution in patients with MS and other autoimmune complications.^13^

***Systemic lupus erythematosus (SLE):*** A 43-year-old woman with RRMS under treatment with INF-β1a was diagnosed with drug-induced SLE (DILE) four weeks after developing synovitis, fever, myalgia, and facial edema. Laboratory test results were positive for antinuclear antibodies (ANA) and anti-double stranded DNA antibody (anti-dsDNA). After discontinuation of INF, the symptoms resolved completely.^14^ A diagnosis of cutaneous SLE was confirmed by lesional skin histopathology from arcuate and polycyclic erythematous scaly plaque on the face, trunk, and upper extremities of a 43-year-old woman with RRMS under treatment with intramuscular (IM) INF-b1a who presented with interface dermatitis.^15^ Lupus erythematosus profundus (lupus panniculitis) was diagnosed when a 19-year-old woman with RRMS under treatment with INF-β1b presented with painful nodules on the face, both shoulders, and upper limbs; a skin biopsy suggested lobular panniculitis.^16^ Two cases of lupus-like reaction to INF at the injection site were reported in patients with MS being treated with INF-β1b.^17^ SLE induced by INF-b1 therapy was reported in a 34-year-old patient with RRMS who developed myalgia associated with wrist synovitis with no skin involvement.^18^

***Psoriasis:*** Case reports have shown an association between psoriasis and MS. In most cases, the psoriasis presented before MS, and it appeared possible to define co-morbidity, but INF-induced psoriasis was not excluded.^19^ Exacerbation of cutaneous psoriasis and psoriatic arthritis has been reported in patients with MS.^20^^-^^22^

***Inflammatory bowel disease (IBD):*** Severe colitis caused by Crohn’s disease was reported in a patient with MS treated with IFN-β.^23^ Aggravation and development of ulcerations was reported after treatment with IFN-β1a.^24^^,^^25^ A case of rapid-onset ulcerative colitis was reported one week after INF-β1a therapy. The time of onset of ulcerative colitis was from one day after IFN initiation to one week after discontinuation of the therapy.^26^ Celiac disease was reported in a 36-year old woman with RRMS after one month of treatment with IFN-β1b.^27^ MS concomitant with celiac disease has been recently diagnosed more often than expected.^27^ A 27-year-old woman with RRMS treated with GA for 2 years presented with abdominal pain, diarrhea, fever, and weight loss. A colonoscopy showed diffuse right-sided colitis and ileitis, and the biopsy was compatible with Crohn’s disease. This report emphasized that Crohn’s disease might be an adverse event related to treatment with GA.^28^

***Sarcoidosis:*** IFN-β has been reported to induce sarcoidosis. The duration of IFN-β treatment at the time of diagnosis varied from 8 months to three years. The most common presentation included pulmonary manifestations, although hepatic, and bone involvement. Transbronchial, mediastinal, and paratracheal lymph node and skin biopsies confirmed the diagnoses.^29^^,^^30^ Cutaneous and pulmonary sarcoidosis with erythema nodosum in the lower limbs, breast abscesses, and unilateral pulmonary adenopathy were reported in a 33-year-old woman with RRMS treated with INF-β1b for 2.5 years.^31^

***Susac syndrome:*** Exacerbation of Susac syndrome retinopathy has been reported in a 23-year-old man misdiagnosed with MS who was treated with IFN-β1a for 15 months. Two weeks after IFN-β discontinuation, the visual field and fluorescein angiography indicated complete recovery.^32^

***Scleromyxedema:*** Scleromyxedema was reported in a 37-year-old woman with RRMS under treatment with IFN-β1a for three years. The patient recovered after discontinuation of IFN-β1a treatment.^33^

***Dermatomyositis:*** A violaceous skin eruption associated with periorbital edema and proximal muscle weakness presented in a 57-year-old man with RRMS under treatment with IFN-β1a. Skin biopsy diagnosed it as dermatomyositis. Clinical exacerbation occurred after restarting IFN, despite the initial improvement after discontinuation.^34^

***Systemic sclerosis*** *:* MS coexisting with systemic sclerosis has been reported.^35^^-^^41^ In most cases, systemic sclerosis presented before MS, but in one case, the systemic sclerosis presented after pulse therapy and initiation of INF in a patient with MS. It has been postulated that high-dose steroids may be a potent predictor of renal crisis, and that IFN may aggravate Raynaud’s phenomenon, and cause vasospasms and vascular occlusion.^42^

***Vitiligo:*** A 33-year-old woman with MS developed depigmented patches on the dorsal aspects of her hands after 2 years of treatment with IFNβ-1a which was diagnosed as vitiligo. After discontinuation of IFN in preparation for becoming pregnant, the size of the lesions decreased, and significant recovery was noted after three months.^43^

***Urticarial vasculitis: ***A 48-year-old woman with RRMS who had been treated for three months with GA showed urticarial-like plaques on her body, face, and extremities which skin biopsy confirmed to be leukocytoclastic vasculitis.^44^

***Anaphylaxis:*** IgE-mediated allergy to INF-β1a was reported in a 34-year-old woman with MS, 15 minutes after injection. It presented with pruritus, urticarial rash, dyspnea, and hypotension, and required emergency hospitalization.^45^ Similar IgE-mediated anaphylaxis has been reported with GA. Two patients presented with generalized urticaria, nausea, and hypotension upon the first administration of GA. In a third patient, after six months of GA interruption, an immediate reaction presented with eyelid edema 20 minutes after GA administration.^46^

***Autoimmune hepatitis (AIH):*** Features of autoimmune liver disease and primary biliary cirrhosis after 33 months of administration of IFN-β was reported in a 42-year-old woman with RRMS. The strong positivity of antimitochondrial antibodies was consistent with primary biliary cirrhosis and positive ANA along with elevated aspartate aminotransferase (AST) and alanine aminotransferase (ALT) were suggestive of AIH; but clinical symptoms of acute liver injury did not present.^47^ A 20-year-old woman and a 47-year-old man with RRMS treated with IFN-β1a presented with elevated liver function tests. The first patient presented with asthenia, progressive jaundice, nausea, and abdominal discomfort along with re-elevated liver function tests despite discontinuation of IFN, and was diagnosed with hepatic encephalopathy. The anti-smooth muscle antibody test was positive. The liver function tests and encephalopathy began to recover after 2 days of corticosteroid therapy. In the second case, 37 months after onset of treatment, elevated transaminase levels presented. After re-introduction of IFN, liver function tests were again elevated. The anti-smooth muscle antibody and antimitochondrial antibody tests were negative, but antithyroperoxidase was positive. A liver biopsy documented severe periportal interface hepatitis. Liver function test results recovered rapidly after corticosteroid therapy.^48^

AIH has also been reported with GA. A patient treated with GA presented with malaise and jaundice along with elevated liver function test after two months of treatment. Although the smooth muscle antibody and antimitochondrial antibody were negative, a significantly elevated titer of 1,280 ANA was detected, and the liver biopsy demonstrated AIH.^49^ A biopsy confirmed AIH after switching treatment to GA. This patient developed jaundice and histologically-documented necrotizing hepatitis. IFN was discontinued and after normalization of liver function tests, GA was initiated; however, liver tests again elevated, and a liver biopsy confirmed AIH. It appears that IFN and GA unmasked AIH.^50^

***Autoimmune hematologic disease:*** A 31-year-old patient with RRMS and a positive Coombs autoimmune hemolytic anemia result presented with fatigue and dizziness two years after starting treatment with INF-β1b. After other possibilities were ruled out, an association with INF was suggested.^51^ A similar scenario has been reported for a 26-year-old woman with RRMS presenting with anemia, jaundice and fatigue 11 months after treatment with INF-β1b. After discontinuation of INF-β1b, the symptoms and abnormal lab test disappeared.^52^

Thrombotic microangiopathy (TMA)-hemolytic uremic syndrome is an unusual side effect of INF. A 37-year-old woman with RRMS and a 20-year history of treatment with INF presented with hypertension, acute renal failure, subnephrotic proteinuria, nausea, and vomiting. A kidney biopsy showed chronic glomerular microangiopathic lesions and moderate interstitial edema with mild inﬂammatory cell infiltration. TMA secondary to INF-β1b was diagnosed. Hematological abnormalities and renal function gradually improved after corticosteroid therapy. 

It appears that IFN participates in endothelial disruption by interruption and uncontrolled activation of complex pathways of complement regulation.^53^ In a 48-year-old woman with RRMS treated with INFβ-1b, hemolytic-uremic syndrome (HUS) was diagnosed after she presented with signs of hypertension, asthenia, and loss of muscular strength in the legs. Creatinine levels increased to 1.8 mg/dl and the platelet (PLT) count decreased to 132 × 10^9^/l. After lack of significant improvement with corticosteroids, she received eculizumab.^54^

A 52-year-old man presented with TMA caused by ADAMTS13 deficiency after treatment with IFN-β.^55^ Three women with RRMS treated with high doses of INFβ-1a were diagnosed with thrombotic thrombocytopenic purpura-hemolytic uremic syndrome (TTP-HUS) when they presented with blurred vision, cephalalgia, confusion, arterial hypertension, seizures, and focal deficits associated with hemolytic anemia and thrombocytopenia.^56^ A 36-year-old patient with RRMS who had been treated with subcutaneous (SC) INF-β1a was hospitalized due to progressive dyspnea, systolic dysfunction and pulmonary hypertension. Laboratory tests revealed anemia, thrombocytopenia, and high lactate dehydrogenase (LDH). A blood smear showed schistocytes, and TMA was suspected.^57^ A 53-year-old woman with RRMS who had been treated with SC INF-β1a for 15 years presented with an epileptic seizure, headaches, confusion, and arterial hypertension. INF-β-associated TMA was diagnosed because of the symptoms of anemia, thrombocytopenia, and elevated LDH, and the presence of schistocytes.^58^ Two cases of INF-associated TMA have been reported, after clinical and laboratory tests showed results similar to the previous scenarios.^59^

Idiopathic thrombocytopenic purpura could be developed secondary to INF-β, and repeated after changing to other forms of INF. In this circumstances, close monitoring of thrombocytopenia is supposed to be considered.^60^

***Nephrologic autoimmune adverse events:*** Glomerulonephritis and nephrotic syndrome was reported in a 40-year-old woman after a long-term INF treatment. Electron microscopy showed immunoglobulin and complement deposits in kidney biopsy. This report suggests that in spite of this INF rare adverse event, physician is supposed to consider of these clinical rare adverse events.^61^

## Discussion

A combination of blood-brain barrier (BBB) breakdown and immune cell activation terminate in demyelination and axonal injury in MS. It appears that drugs similar to IFN and GA modulate and reduce this inflammatory process. The precise INF and GA mechanisms of action are unclear. Binding of INF-β to its receptors reduces antigen-presenting cells and T-cell proliferation. Its effect on cytokine and matrix metalloproteinase expression potentiates suppressor functions.^62^ IFN-ƴ antagonism, stop of immune-cell trafficking across the BBB, autoreactive T-cell apoptosis, and induction of anti-inflammatory cytokine shifts have also been theorized as potential mechanisms of INF-β action.^63^ Induction of T-helper 17 (Th17) cells which may be involved in MS lesions through downregulation of its inflammatory response pathways is another proposed INF-β mechanism of action.^64^ Some evidence of a pro-inflammatory role of INF-β, including an increase in interleukin-6 (IL-6) production in central nervous system astrocytes,^65^ an anti-apoptotic effect on T-cells,^66^ and activation of antigen-presenting dendritic cells,^67^ have been reported.

Several studies showed that a protective action of GA presenting as a switch from Th1 to Th2 (anti-inflammatory cytokine secretion) type responses.^68^ Apart from the effect of GA on CD4+ T-cells, it could cause CD8+ T-cell upregulation, and enhance suppressor activity of CD8+ T cells.^69^ GA could affect B-cells, and might affect differentiation and polarization of T-cells. GA-activated B-cells enhance secretion of anti-inflammatory cytokines such as IL-10, IL-4, and transforming growth factor beta (TGF-β).^70^ GA has caused notable elevation in IgG4 antibody titers with low pro-inflammatory effects,^71^ and increased IgG1 antibodies levels more than IgG2.^72^ On the other hand, antibodies to GA do not affect GA biological activity, as is similar in INF-β.^73^ GA enhances a natural killer cell cytolytic effect against autologous and allogeneic mature and immature monocyte-derived dendritic cells.^74^

Of 10 comparative studies, only two reported a higher prevalence of rheumatoid arthritis in patients with MS than in matched control groups; eight studies reported no differences.^75^ It appears that the reports of autoimmune arthritis in patients with MS after treatment with INF-β and GA are more pronounced than autoimmune arthritis co-occurrence with MS. The results are similar for SLE.^75^ The incidence of SLE was not significantly different from expectations for the general population. Of five studies, only one showed an increased incidence of SLE in patients with MS.^75^ The incidence and prevalence studies of psoriasis in the MS population showed no meaningful differences with the general population; only one study reported increased prevalence of psoriasis in MS population when compared with the control population.^75^ Most studies reported increased incidence prevalence of IBD before and after of MS diagnosis when compared with the general population. The incidence and prevalence of autoimmune hematologic disease also showed no significant differences between the MS population and a matched control population.^75^ A Danish study reported the prevalence of dermatomyositis in patients with MS to be zero. Its prevalence in other studies was 0.20% to 0.03%. Studies on the incidence and prevalence of vitiligo and AIH in the MS population found no significant differences with control populations.^75^

A recent systemic review documented no increase in risk of co-morbid autoimmune disease with MS. Fewer than 5% of individuals with MS are affected by co-morbid autoimmune diseases. Shared environmental and genetic susceptibility or both may explain this co-occurrence.^75^ These findings suggest that the pro-inflammatory role of INF-β figures in the presentation of autoimmunity during treatment. In reality, there is a balance between the pro-inflammatory and anti-inflammatory roles of INF-β. Genetic, and immunological and environmental susceptibility may contribute to the pro-inflammatory role in the appearance of a new autoimmune disease. INF composed of highly immunogenic proteins which act on immune system. Flu-like syndrome after the first hours of INF administration defines as immediate reactions, and probably appears due to cytokines release by immune cells. Delayed hypersensitivity can present between 1-2 hours and 14 days after administration. A serum sickness or Arthus-like reactions might be mediated by soluble immune complexes of INF with its antibodies. This type III hypersensitivity reaction may be the pathophysiology of some autoimmune adverse events of INF such as arthritis.^76^^-^^78^

The case reports suggest that if symptoms, signs, or laboratory findings indicate possible autoimmunity in patients with MS, it is better to select GA for treatment over INF-β. The case reports demonstrate that there are fewer adverse autoimmune events related to GA than INF-β. One reason has been shown to be the immunomodulatory effects of GA on autoimmune diseases. Studies indicate that GA decrease experimental autoimmune uveoretinitis,^79^ and has demonstrated a therapeutic effect on IBD.^80^^,^^81^ GA may prevent graft-versus-host disease,^82^ and have a potentially therapeutic effect on type 1 diabetes.^83^ GA has showed no beneficial effects on a spontaneous model of SLE,^84^ and even exacerbated collagen-induced autoimmune arthritis.^13^

## Conclusion

Although co-morbid autoimmune disorders have been reported in patients with MS, the pro-inflammatory role of disease-modifying drugs, especially INF-β, could affect and enhance this co-occurrence. A clinical or laboratory autoimmunity history suggest the use of GA over INF-β as the treatment of choice.

## Notes:

**How to cite this article:** Baghbanian SM, Sahraian MA. Induction or aggravation of other immune-mediated disorders by disease-modifying therapy in treatment of multiple sclerosis. Iran J Neurol 2018; 17(3): 129-36.
